# The paradigm of tumor shrinkage and rapid liver remnant hypertrophy for conversion of initially unresectable colorectal liver metastasis: a case report and literature review

**DOI:** 10.1186/s12957-017-1212-6

**Published:** 2017-08-03

**Authors:** Nan Xiao, Kailin Yu, Shaojun Yu, Jianjun Wu, Jian Wang, Siyang Shan, Shuchun Zheng, Liuhong Wang, Jianwei Wang, Shuyou Peng

**Affiliations:** 1 Department of Surgical Oncology, Second Affiliated Hospital of Zhejiang University School of Medicine, Hangzhou, 310009 China; 2 Department of Radiology and Intervention, Second Affiliated Hospital of Zhejiang University School of Medicine, Hangzhou, China; 3 Department of General Surgery, Second Affiliated Hospital of Zhejiang University School of Medicine, Hangzhou, China

**Keywords:** Tumor shrinkage, Liver remnant hypertrophy, Unresectable colorectal liver metastasis, Preoperative chemotherapy

## Abstract

**Background:**

For colorectal liver metastasis (CRLM) patients, hepatic resection is currently the sole cure offering the chance of long-term survival. Tumor shrinkage and planned liver remnant hypertrophy are the two key strategies for conversion of initially unresectable CRLM. First conducted in 2012, associated liver partition and portal vein ligation for staged hepatectomy (ALPPS) allows rapid liver growth. As a means to induce hypertrophy, portal vein embolization (PVE) has been widely applied before extending hepatectomy. Recently, Peng et al. present a new approach of terminal branches portal vein embolization (TBPVE), offering an efficient way to amplify FLR and making chances for surgery in 2 weeks.

**Case presentation:**

We reported a 61-year-old woman with synchronous hepatic metastasized carcinoma of the colon sigmoideum underwent TBPVE after 6 cycles of neoadjuvant therapy in order to perform a planned right trisectionectomy. Rapid liver remnant hypertrophy and remarkable tumor shrinkage were achieved, and laparoscopic sigmoidectomy and right trisectionectomy were successfully performed. The postsurgical course was uneventful and 7 months of recurrence-free survival have been witnessed.

**Conclusions:**

The dual tactics of tumor shrinkage and planned rapid liver remnant hypertrophy will make concerted efforts to further increase the clinical candidacy for curative resection, which are valuable for further investigation.

## Background

Colorectal cancer is the third leading cause of malignancy-related death worldwide. The liver is the most common metastatic site of colorectal cancer and liver metastasis occurs in 20–40% of patients at the time of primary diagnosis [1]. Liver resection is the sole curative approach to colorectal liver metastasis (CRLM). Nevertheless, only less than 25% of patients with CRLM are eligible for curative resection, of which insufficient future liver remnant (FLR) volume is the main cause [2]. Currently, systemic therapy is the only established treatment for patients with unresectable CRLM. Tumor shrinkage and liver remnant hypertrophy are the two key strategies for conversion of initially unresectable CRLM.

First conducted in 2012, associated liver partition and portal vein ligation for staged hepatectomy (ALPPS) allows rapid liver growth in a short time and has a strong impact on surgery for liver cancer. However, high morbidity and mortality after the surgery cannot be ignored. Classic portal vein embolization (PVE) has been widely applied before extending hepatectomy, but the slow growth of FLR renders a longish waiting time of 4–6 weeks between PVE and liver resection, which embraces the risk of tumor progression. Recently, Peng et al. [3] presented a new approach of terminal branches portal vein embolization liver partition for planned hepatectomy (TELPP), offering an efficient way to amplify the FLR and making chances for surgery in 2 weeks.

## Case presentation

A 61-year-old woman was referred to our department because of synchronous hepatic metastasized carcinoma of the colon sigmoideum. Physical examination revealed no significant abnormalities. Blood investigations were within normal range except for mild anemia and serum carcinoembryonic antigen level of 10 ng/ml. Fecal occult blood test was strongly positive.

**Fig. 1:**
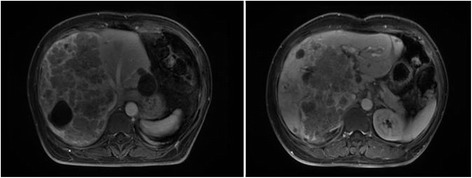
MRI imaging of the mass revealed tumor distributed at the right hepatic trisection, adjacent to the left branch of portal vein at primary diagnosis

Colonoscopy found a sigmoid lesion 20 cm from the anus, and pathological result of the biopsy specimen demonstrated a moderately differentiated adenocarcinoma. The gene detection found no mutation in KRAS, NRAS, and BRAF. Magnetic resonance imaging of the liver revealed multiple metastases and the largest measured 15 cm × 11 cm × 13 cm (staging according the seventh edition of the UICC: cT3, cN1, cM1a) (Fig. 1).

The multi-disciplinary team (MDT) suggested a conversion strategy consisting of preoperative FOLFIRI (irinotecan, fluoruracil) regimen with cetuximab for the initially unresectable colorectal liver metastasis. If the conversion of liver metastasis was achieved, a right trisectionectomy would be performed.

**Fig. 2:**
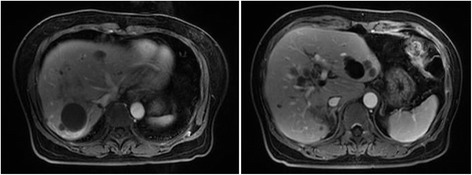
Tumor shrunk significantly after 6 cycles of FOLFIRI with cetuximab

**Fig. 3:**
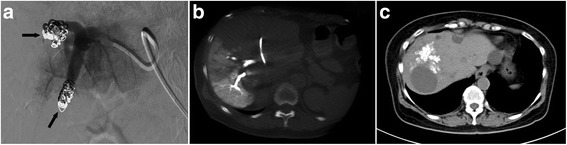
Effect of terminal branch portal vein embolization. **a** Post-embolization venogram with coils visible in the anterior and posterior right portal vein (*black arrows*). **b** CT scan showing embolized terminal branches of the right portal vein. **c** CT scan showing lipiodol deposition

After 6 cycles, the liver metastasis significantly shrank (staging according to RECIST version 1.1: PR), but the largest metastasis was adjacent to the left branch of portal vein (Fig. 2). The CT volumetry revealed that total liver volume (TLV) was 1495 ml, and the volume of the future liver remnant (FLR) was 415 ml, which was below the volume cutoff value for safe resection (more than 40% of TLV) [4]. So, according to MDT, terminal branches portal vein embolization (TBPVE) was performed to block the anterior and posterior branch of the right portal vein as previously reported [3] for sufficient hypertrophy of the left lateral lobe (Fig. 3).

**Fig. 4:**
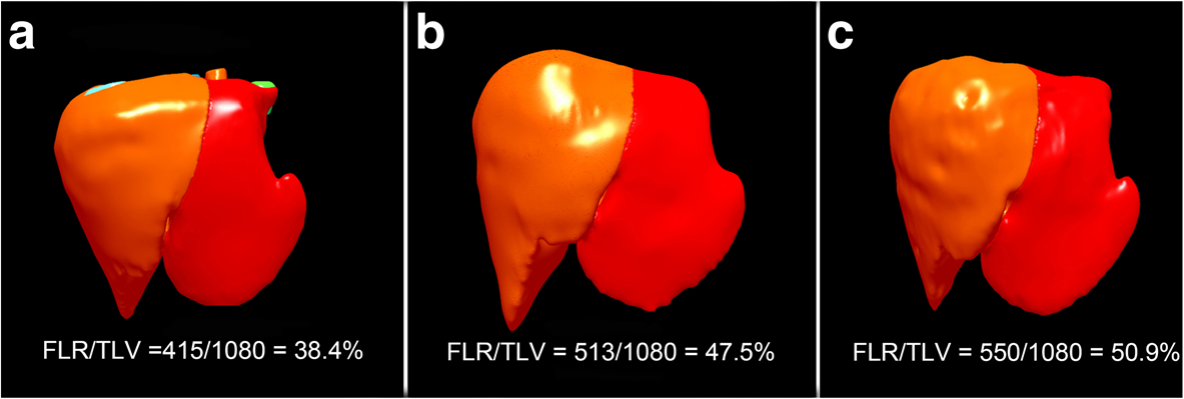
Volume of the left lateral external lobe of the liver. **a** Before the TBPVE. **b** Hypertrophy on the 5th and **c** 11th day after the TBPVE

**Fig. 5:**
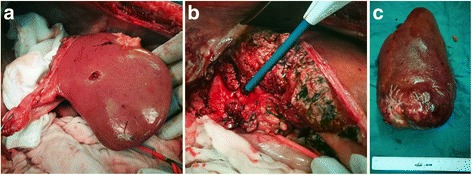
Right trisectionectomy **a** hypertrophy of the left lateral external lobe of the liver **b** pars sagitalis of the left branch of portal vein **c** right trisectionectomy specimen

Five days after the TBPVE, the FLR was 513 ml (47.5% of TLV). On the 11th day, the FLR was 550 ml (50.9% of TLV) (Fig. 4) and laparoscopic sigmoidectomy and right trisectionectomy were performed (Fig. 5). There were no intraoperative complications, and the histology of the FLR showed a low-grade steatohepatitis after chemotherapy. The postoperative histology revealed pT3, pN2b (11/17), pM1, L0, V1, G2, and R0 resection margin. The postsurgical course was uneventful and the patient continued to undertake 6 cycles of systemic therapy of FOLFIRI with cetuximab. Heretofore, 7 months of recurrence-free survival have been witnessed.

## Discussion

For CRLM patients, hepatic resection is the only treatment with a curative intent offering the chance of long-term survival at present. Nevertheless, at the time of diagnosis, only fewer than 25% of CRLM patients are eligible for liver resection. The main limitations to the resection are inadequate future liver remnant (FLR) volume and poor oncological prognosis of advanced diseases. The former increases the risk of postoperative liver failure, while the latter is associated with early recurrence.

The past few decades have seen surgeons struggling to achieve rapid liver remnant hypertrophy. As a classic way to induce hypertrophy of the FLR, PVE is now routinely applied to improve the rate of R0 resection. But it takes 3–8 weeks to expand the volume of FLR by up to 40% and the second stage of the surgery is not always accomplished [5]. First performed by Schnitzbauer in 2012, ALPPS led to 74% increase of the FLR in a mean time of 9 days, making initially unresectable hepatic lesions resectable. Despite its advantages, ALPPS caused high morbidity (64%) and liver-related mortality (12%), which has been evoking violent controversy [6].

To overcome the aforementioned disadvantages, Peng et al. [3] presented a new approach of TBPVE, which deflowered the essence of PVE and ALPPS. It is reported that this new technique could induce rapid liver remnant hypertrophy and make chances for surgery in 14 days. Increasing FLR by 33% to 68%, four cases of huge hepatocellular carcinoma with liver cirrhosis acquired chances of right hepatectomy 2 weeks after the TBPVE. His group recently reported the safety and efficacy data of tentative application of TBPVE liver partition for planned hepatectomy (TELPP). Similar to that of ALPPS, the philosophy behind TBPVE is the thorough separation of the left and right hemi-liver (ramus communicans of the right and left portal vein). Moreover, complete and enduring embolism of the terminal branches to segment V, VIII or IV is indispensible in order to rapidly promote the regeneration of remnant liver parenchyma. Technically, to achieve this goal, they first embolized the terminal branches with lipiodol and cyanoacrylate, then blocked the main branch of portal vein with coils. Compared with classic PVE, the new technique could increase the FLR more significantly in a short period of time. The specific physiopathological mechanisms underlying this enhanced liver remnant hypertrophy are still unconfirmed. The explanation for this phenomenon might be that, in comparison to PVE, TBPVE attains more thorough partitions of communicating branches between the planned resected segments and FLR, which lead to increasing portal flow and hepatotropic factors to the FLR [7].

The use of first-line chemotherapy to shrink hepatic metastases is an ideal strategy for patients with initially unresectable CLRM, but only about 20% of the patients with initially unresectable liver-limited metastases become resectable after chemotherapy [8]. Considering the low tumor response rate yielded by second-line chemotherapy after the failure of first-line regimen [9], we believe TBPVE could be a better choice for these patients as a fast and efficient way to amplify FLR and create chances for surgery in a short time.

There is growing evidence that PVE not only does stimulate the growth of FLR but also induces significant tumor growth in patients with CRLM [10, 11]. The longish time between PVE and liver resection required to achieve adequate FLR volume embraces the risk of progressive disease [12]. Moreover, potential promotion of tumor growth after PVE and consequent acceleration of tumor progression in the waiting time are primary concerns possibly confining the use of PVE in patients with multifocal tumors [13]. A recent meta-analysis [5] reviewing 44 publications and including 1791 patients undergoing PVE demonstrated that the hypertrophy response was insufficient in 51 patients (2.8%) to perform liver resection and 6.1% of those patients treated ultimately were not able to undergo resection because of local tumor progression after PVE. Another study by Fischer et al. [14] based on 208 tumors measured in 64 patients found that, without post-PVE chemotherapy, 34.2% of the liver lesions progressed. However, there was a remarkable lower risk of tumor progression (18.9%, *p* = .03) when chemotherapy was applied.

Consequently, it has sparked controversy over whether preoperative chemotherapy could cooperate with PVE.

Surely, there are sufficient practical and academic justifications for preoperative chemotherapy. Approximately 15% of patients with initially unresectable hepatic colorectal metastases are now conventionally converted to resectable cases by chemotherapy. The chemotherapy and biologic treatments given while the tumor is in situ can not only help to decide the appropriate therapies after surgery but also eliminate microscopic tumor cells.

Nevertheless, it was suggested that preoperative chemotherapy would hinder liver regeneration [15] and increase postoperative complications [16]. Therefore, chemotherapy was interrupted several weeks before embolization, leaving the potential for tumor progression at liberty.

Prolonged peri-procedure chemotherapy has been associated with reduced hypertrophy [17, 18]. Yet, some studies [19–21] were unable to show any influence of chemotherapy on liver regeneration after PVE. On the other hand, Covey et al. [22] in a study with a series of 100 patients concluded that liver could still hypertrophy in a toxic environment and preoperative chemotherapy during PVE had no negative effects on liver regeneration. Most recently, Fischer et al. [14] reported the combination of PVE and chemotherapy was not only effective in terms of liver hypertrophy but also related to retarded tumor growth and improved long-term survival. Additionally, it is indicated that short chemical-free intervals (CFI) improved outcomes, whereas long CFI resulted in poor oncological endings. Kambakamba et al. [23] found that short CFI is closely associated with significantly better prognosis in terms of overall survival and disease-free survival based on 74 patients suffering from CRLM who received operations. Spelt and his colleagues [24] also came to the conclusion that long intervals between the end of chemotherapy and PVE could enhance tumor progression in CRLM patients.

## Conclusion

In conclusion, TBPVE was a promising approach to shorten the waiting time between embolization and surgery. Preliminary study indicated that TBPVE could rival ALPPS in respect of inducing rapid liver remnant hypertrophy and, meanwhile, be on a par with PVE in terms of post-operation complications. To confirm its safety and efficacy, further large-scale and multi-centered studies are needed.

The threat of progressive disease after PVE highlights the value of minimizing the waiting time between PVE and resection and of devising therapeutic strategies using preoperative chemotherapy to control tumor growth after PVE. Besides, given that the liver can still regenerate when cytotoxic chemotherapy is administered, the modality combining preoperative chemotherapy with TBPVE would attain lesion shrinkage while also achieving rapid liver remnant expansion. The dual tactics of tumor shrinkage and planned rapid liver remnant hypertrophy will make concerted efforts to further increase the clinical candidacy for curative resection, which are valuable for further investigation.

## Abbreviations

ALPPS: Associated liver partition and portal vein ligation for staged hepatectomy
CFI: Chemical-free intervals
CRLM: Colorectal liver metastasis
FLR: Future liver remnant
MDT: Multi-disciplinary team
PVE: Portal vein embolization
TBPVE: Terminal branches portal vein embolization
TELPP: Terminal branches portal vein embolization liver partition for planned hepatectomy
TLV: Total liver volume
